# Supplementary material for: Peelable Alginate Films Reinforced by Carbon Nanofibers Decorated with Antimicrobial Nanoparticles for Immediate Biological Decontamination of Surfaces
Source: Nanomaterials (Basel). 2023 Oct 16;13(20):2775. doi: 10.3390/nano13202775 (PMC10609245; doi:10.3390/nano13202775)
Supplement: Supplementary file 1 [file nanomaterials-13-02775-s001.zip › nanomaterials-2574642-supplementary.pdf]

Supplementary materials

Peelable Alginate Films Reinforced by Carbon Nanofibers Decorated with Antimicrobial Nanoparticles for Immediate Biological Decontamination of Surfaces

Gabriela Toader [1,†], Aurel Diacon [1,2,†], Edina Rusen [2,*], Ionel I. Mangalagiu [3,*], Mioara Alexandru [4], Florina Lucica Zorilă [4,5], Alexandra Mocanu [2,6], Adina Boldeiu [6], Ana Mihaela Gavrilă [7], Bogdan Trică [7], Daniela Pulpea [1], Mădălina Ioana Necolau [2,8] and Marcel Istrate [9]

[1] Military Technical Academy "Ferdinand I", 39-49 G. Cosbuc Blvd., 050141 Bucharest, Romania
[2] Faculty of Chemical Engineering and Biotechnologies, University Politehnica of Bucharest, 1-7 Gh. Polizu Street, 011061 Bucharest, Romania
[3] Faculty of Chemistry, Alexandru Ioan Cuza University of Iasi, 11 Carol 1st Blvd., 700506 Iasi, Romania
[4] Microbiology Laboratory, Horia Hulubei National Institute for R&D in Physics and Nuclear Engineering, 30 Reactorului St., 077125 Bucharest, Romania
[5] Department of Genetics, Faculty of Biology, University of Bucharest, 91-95 Splaiul Indepententei, 050095 Bucharest, Romania
[6] National Institute for Research and Development in Microtechnologies—IMT Bucharest, 126A Erou Iancu Nicolae Street, 077190 Bucharest, Romania; adina.boldeiu@imt.ro
[7] National Institute of Research and Development for Chemistry and Petrochemistry, 202 Splaiul Independentei, 060041 Bucharest, Romania
[8] Advanced Polymer Materials Group, University Politehnica of Bucharest, 1-7 Polizu Street, 011061 Bucharest, Romania
[9] S.C. Stimpex S.A., 46-48 Nicolae Teclu Street, 032368 Bucharest, Romania

† These authors contributed equally to this work;

*Correspondence to: edina_rusen@yahoo.com and ionelm@uaic.ro;

Table of Contents

Figure S1 – TEM images for silver nanoparticles 2
Figure S2 – EDX spectra of CNF-ox-AgNPs 3
Figure S3 – TEM images for copper nanoparticles 4
Figure S4 – EDX spectra of CNF-ox- Cu-Cu_2O - NPs 5
Figure S5 – TEM images for zinc oxide nanoparticles 6
Figure S6 – EDX spectra of CNF-ox- ZnO - NPs 7
Table S1 - Bacterial cell population decrease after 24h of contact with nanoparticle solutions 7
Figure S7- MIC determination for the synthesized antimicrobial nanomaterials 8
Figure S8 - MIC determination for CNF-ox 9
Figure S9 - MIC determination for the decontaminating formulations 9
Figure S10– Images of the hydrogel before and after peeling 10
Figure S11 – Images of the decontamination solutions and samples for tensile testing 10
Figure S12 – Images of the hydrogels during mechanical properties characterization 10

Supplementary materials

Figure S13 DLS analysis for the nanoparticles and CNF-ox decorated with nanoparticles......... 11
Table S2 - Zeta (ζ) potential measurements results 12

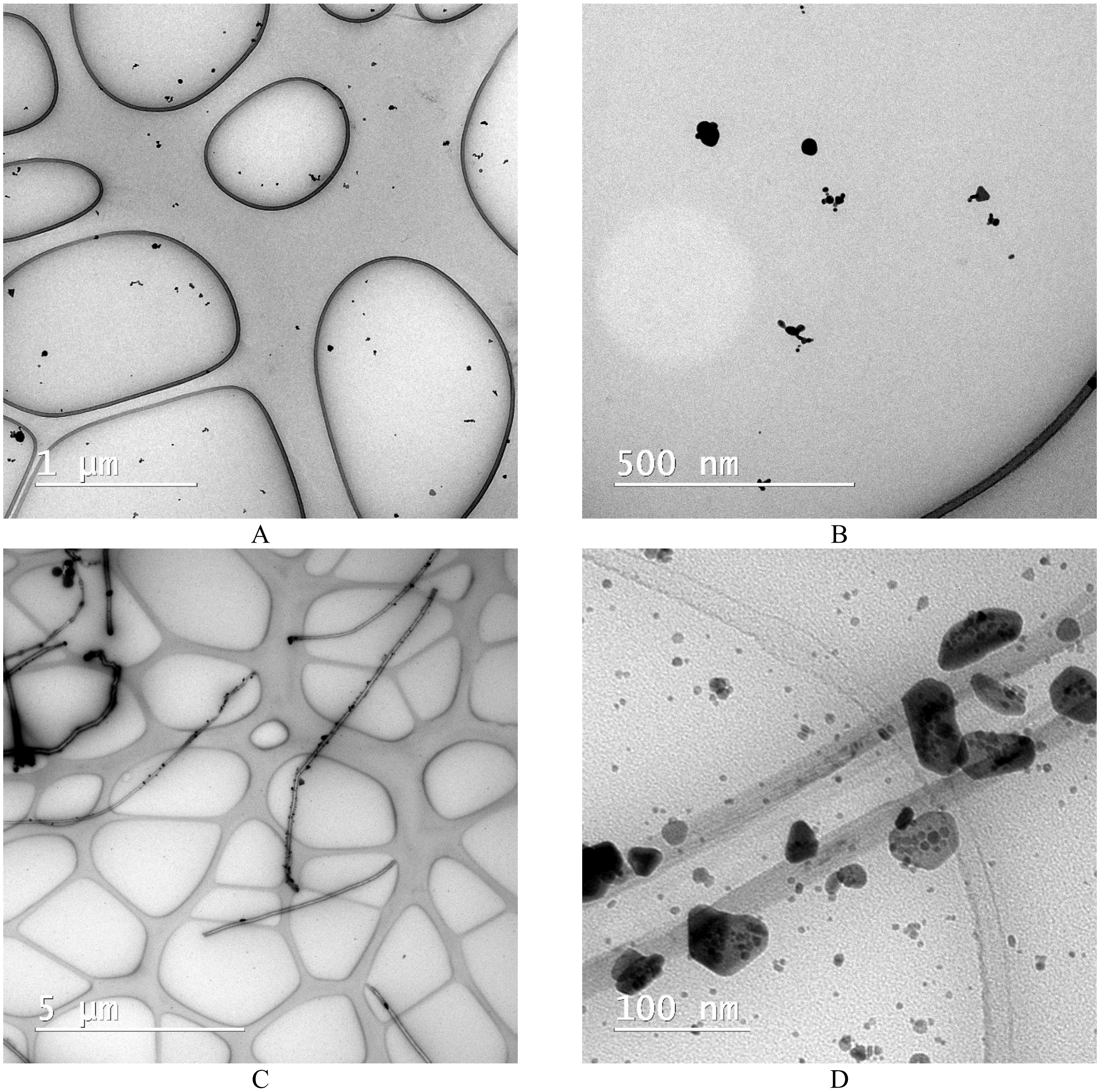

Figure S1 – TEM images for silver nanoparticles
(A, B): AgNPs; (C, D): CNF-ox-AgNPs

Supplementary materials

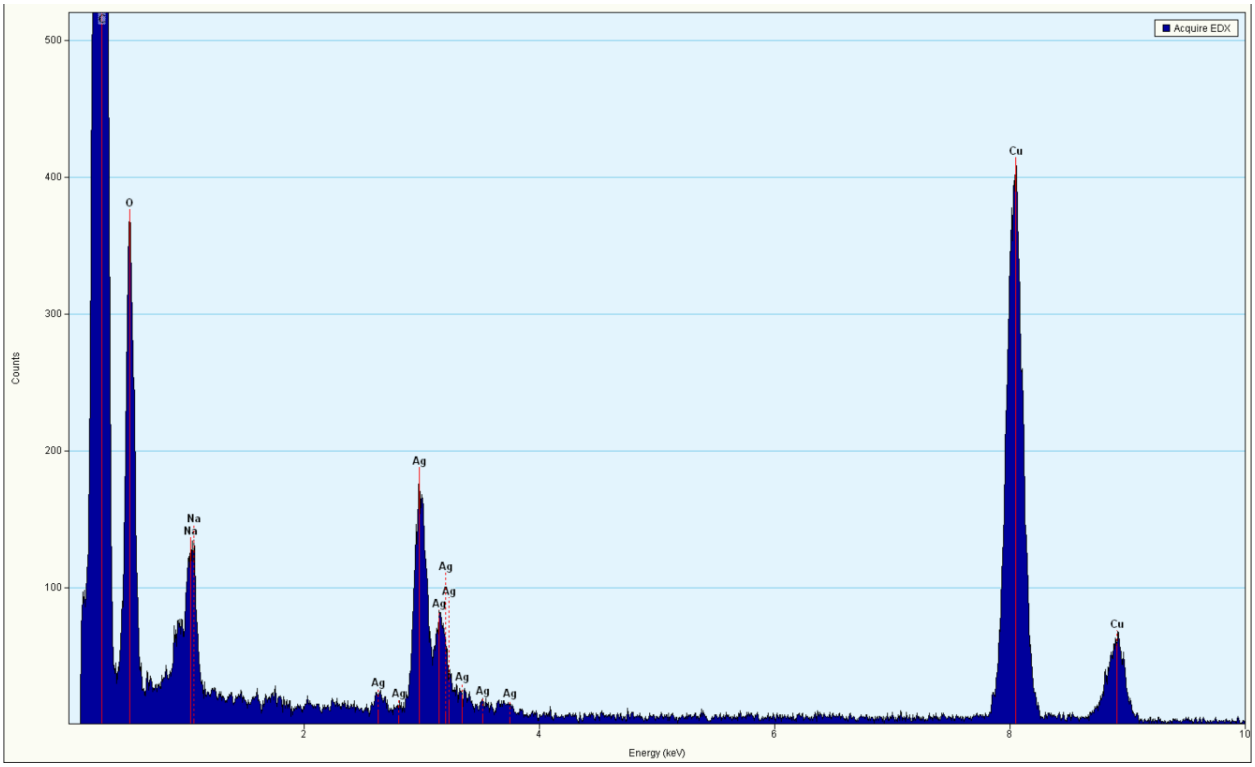

Figure S2 – EDX spectra of CNF-ox-AgNPs

Supplementary materials

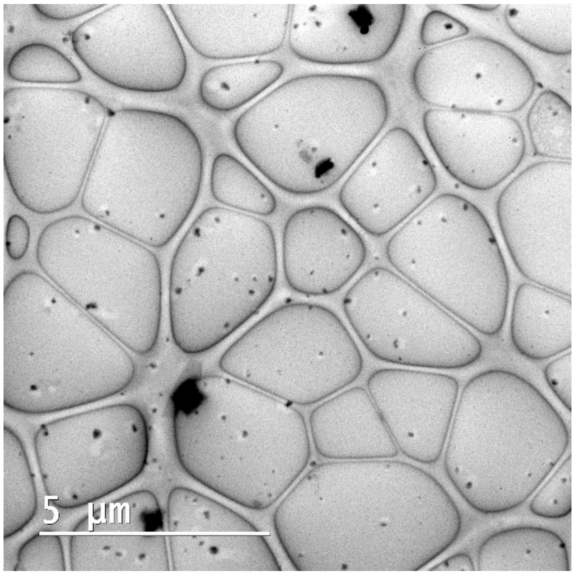

A

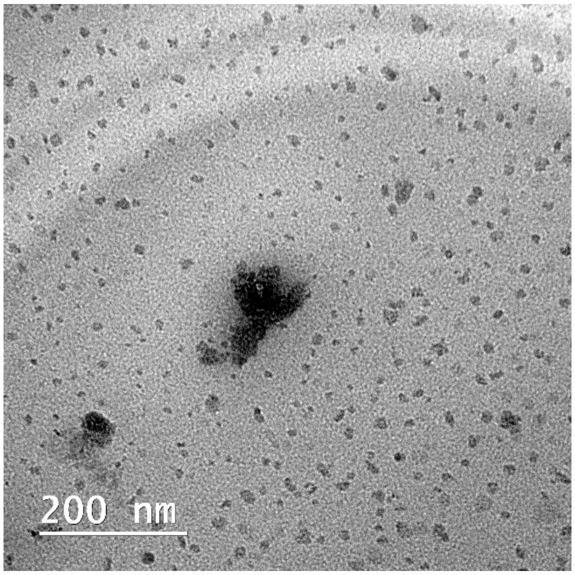

B

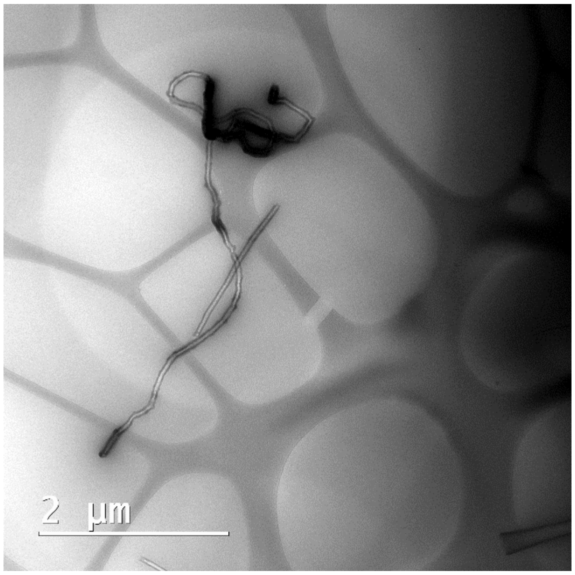

C

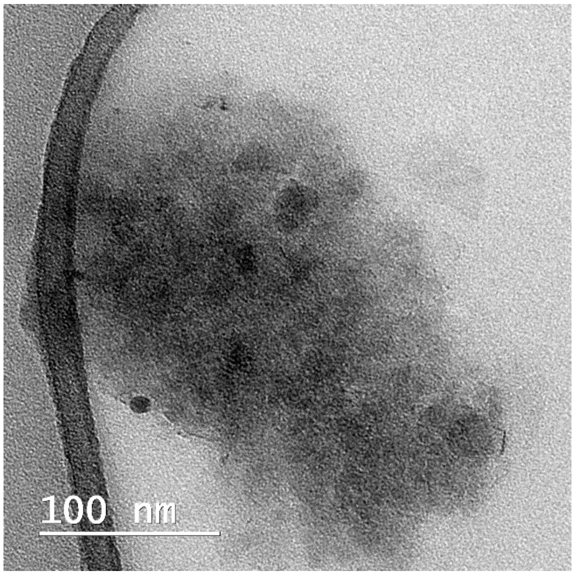

D

Figure S3 – TEM images for copper nanoparticles

(A, B): Cu-Cu_2O - NPs;

(C, D): CNF-ox-Cu-Cu_2O - NPs;

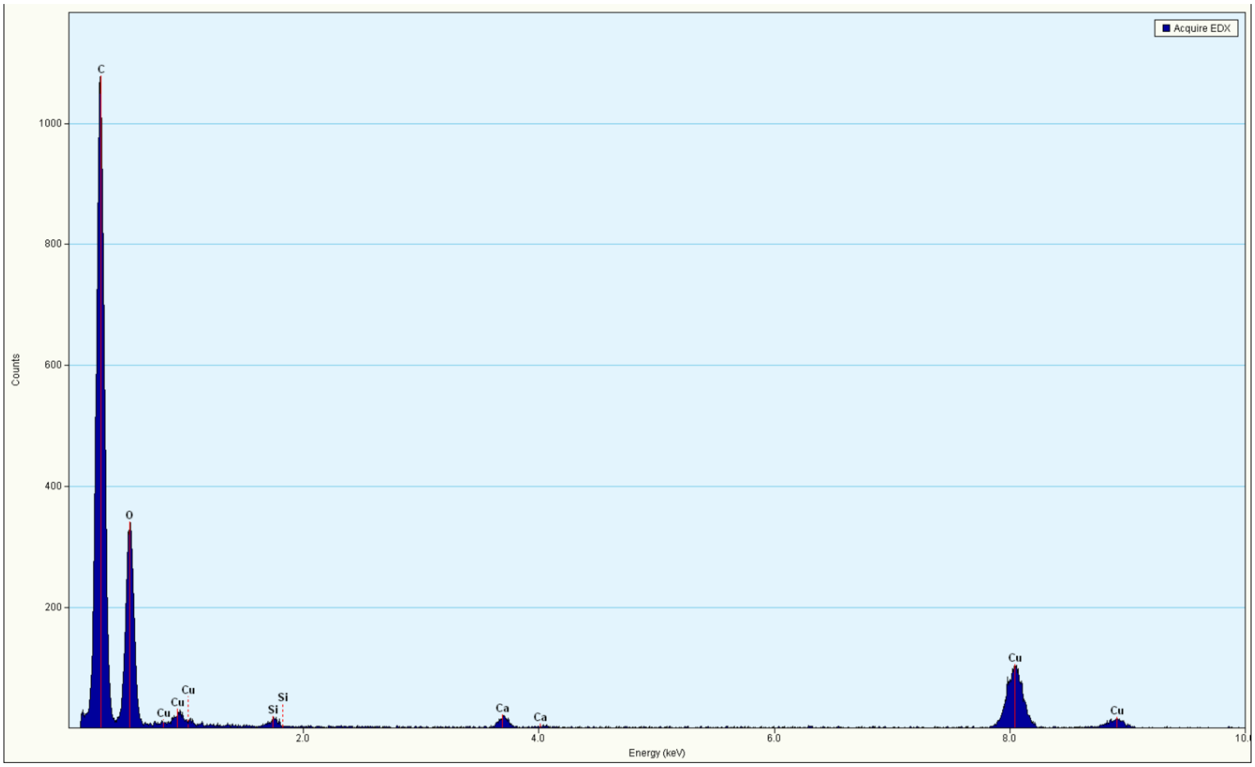

Figure S4 – EDX spectra of CNF-ox- Cu-Cu_2O - NPs

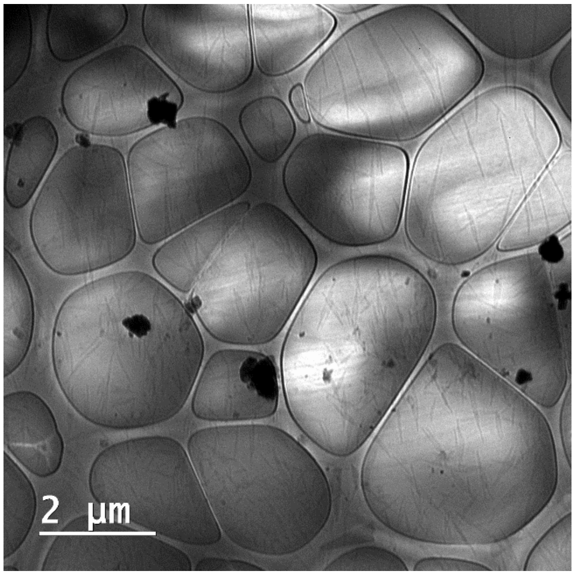

A

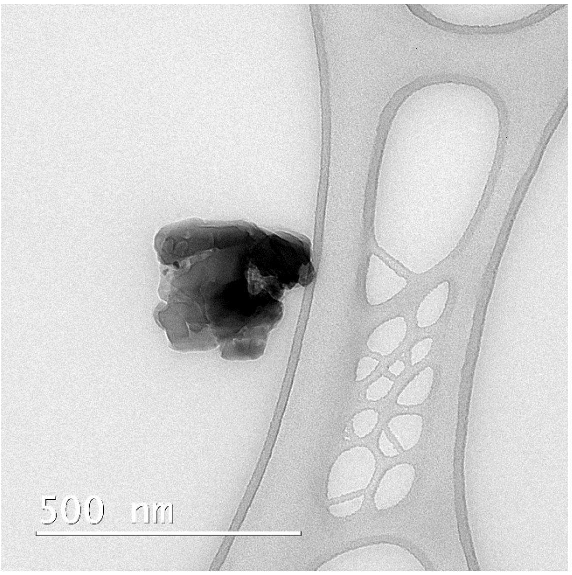

B

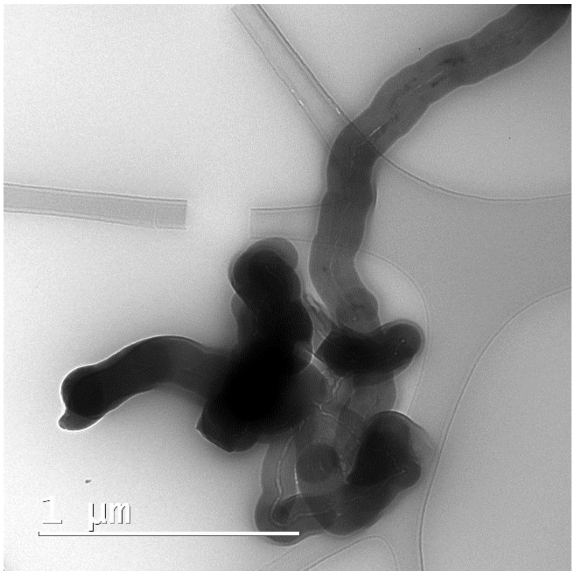

C

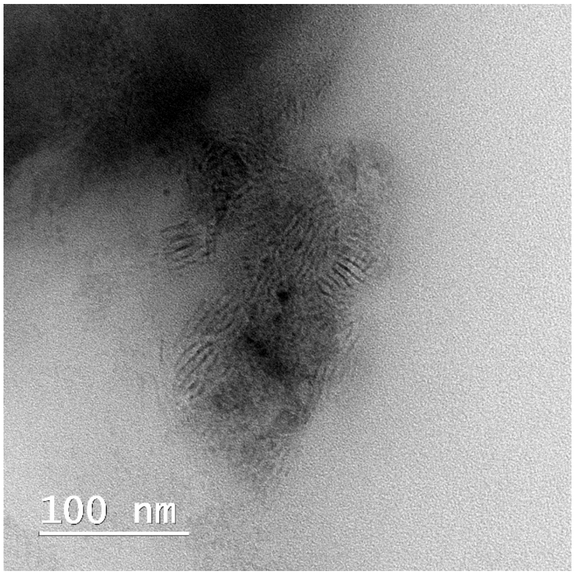

D

Figure S5 – TEM images for zinc oxide nanoparticles

(A, B): ZnO - NPs;

(C, D): CNF-ox-ZnO - NPs;

Supplementary materials

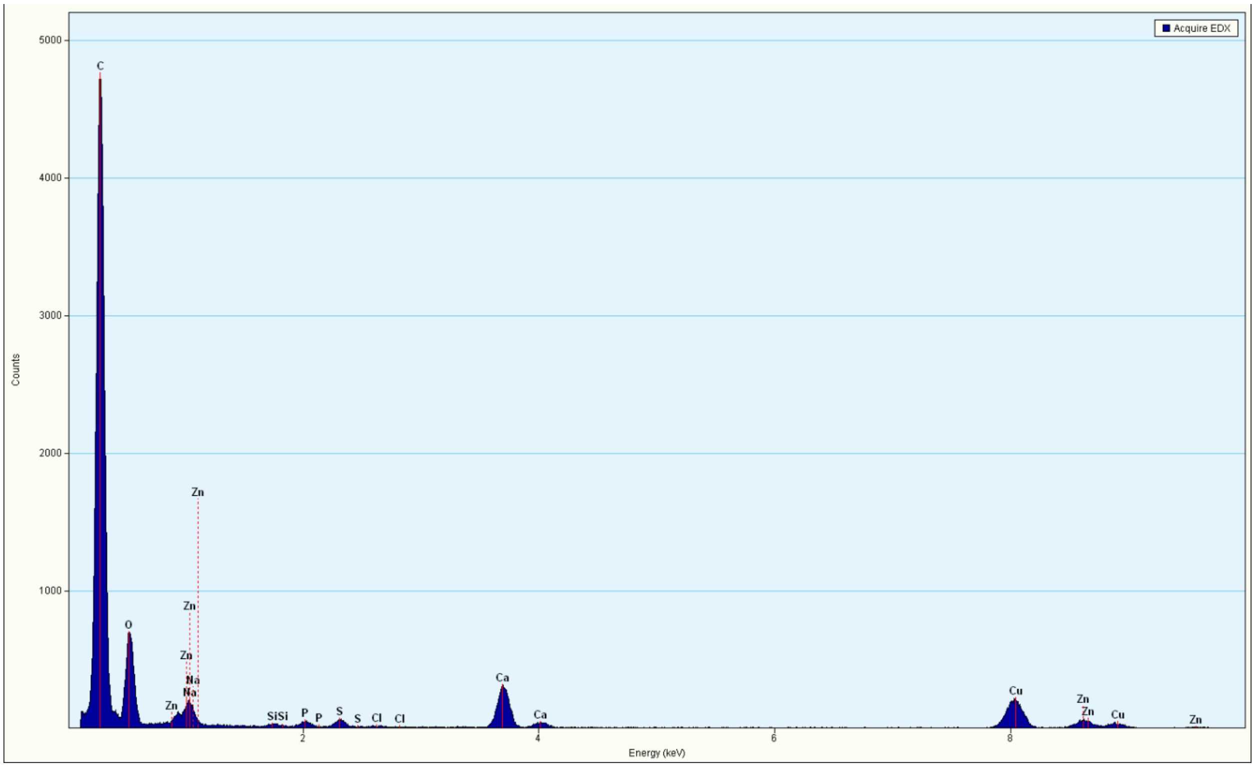

Figure S6 – EDX spectra of CNF-ox- ZnO - NPs

Table S1 - Bacterial cell population decrease after 24h of contact with nanoparticle solutions

Microorganism/ Antimicrobial nanoparticles	ZnO-NPs (%)	CNF-ox-ZnO (%)	Ag-NPs (%)	CNF-ox-Ag (%)	Cu-NPs (%)	CNF-ox-Cu (%)	CNF-ox (%)	CP (%)
E. coli	99.99	99.99	99.99	99.99	99.99	99.99	5.26	7.89
S. aureus	99.99	99.99	99.99	99.99	99.99	99.99	15.38	23.07
P. aeruginosa	99.99	99.99	99.99	99.99	99.99	99.99	46.43	32.14

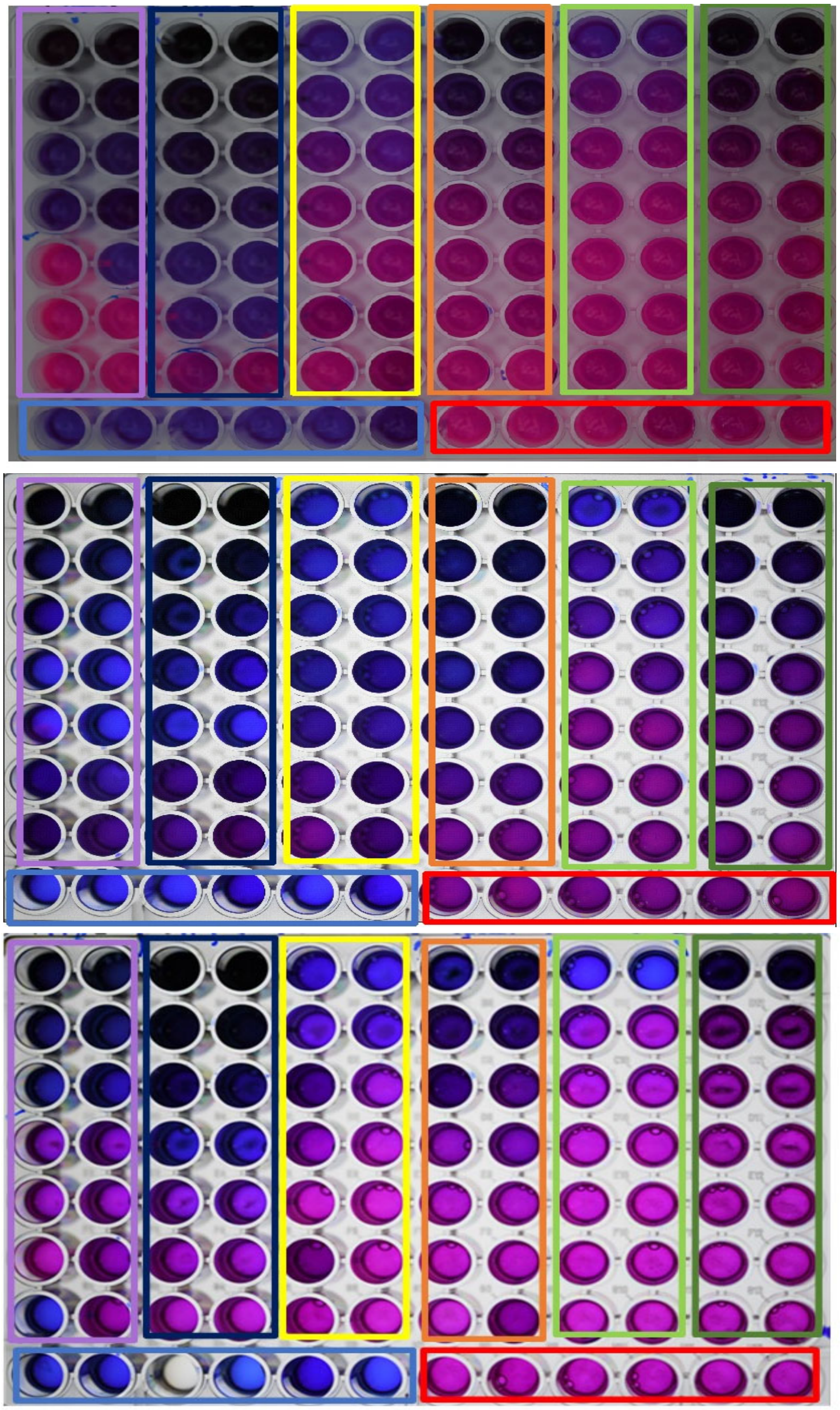

A

B

C

Figure S7- MIC determination for the synthesized antimicrobial nanomaterials
observed from broth microdilution assay using MH broth and resazurin against:
E. coli ATCC 8739 (**A**), *P. aeruginosa* ATCC 9027 (**B**) and *S. aureus* ATCC 6538 (**C**)
Ag-NPs; CNF-**ox-Ag**; **Cu-NPs**; **CNF-OH-Cu**; **ZnO- NPs**; **CNF-ox-ZnO**
Negative Control; Positive **Control**

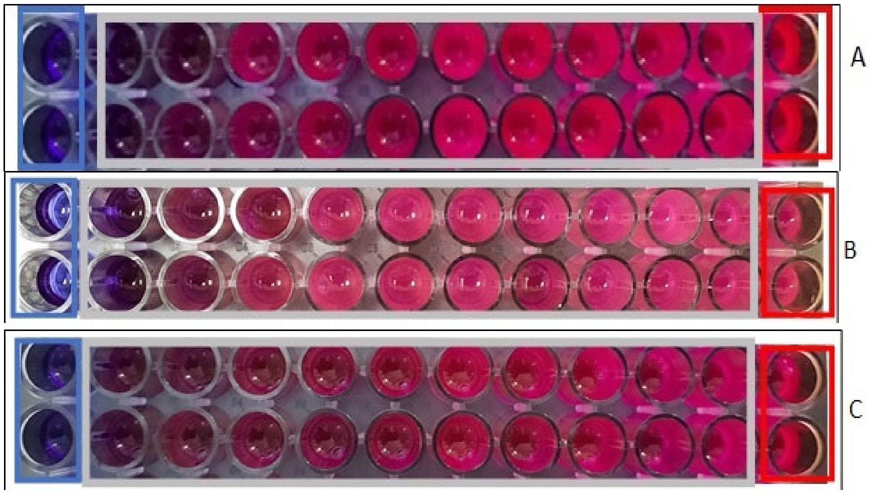

Figure S8 - MIC determination for CNF-ox
observed from broth microdilution assay using MH broth and resazurin against:
E. coli ATCC 8739 (**A**), *P. aeruginosa* ATCC 9027 (**B**) and *S. aureus* ATCC 6538 (**C**)
Negative Control; **BK-CNF**; **Positive Control**

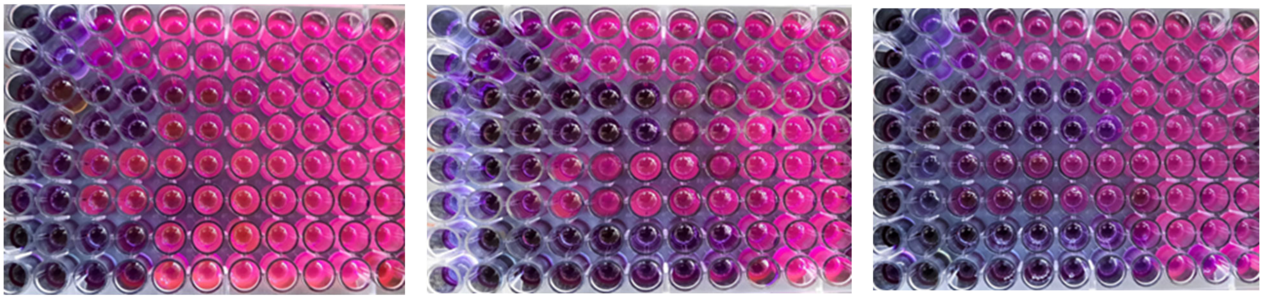

A B C

Figure S9 - MIC determination for the decontaminating formulations
against:

A) *S. aureus* ATCC 6538; B) *E. coli* ATCC 8739 and C) *P. aeruginosa* ATCC 9027
observed from broth microdilution assay using MH broth and resazurin

rows:
A-B = PVA-ALG; **C-D** = PVA-ALG -Ag; **E-F** = PVA-ALG-c2-CNF-OH; **G-H** = PVA-ALG-c2-CNF-OH -Ag;
columns:
1 = Bk, column 11= PC

Supplementary materials

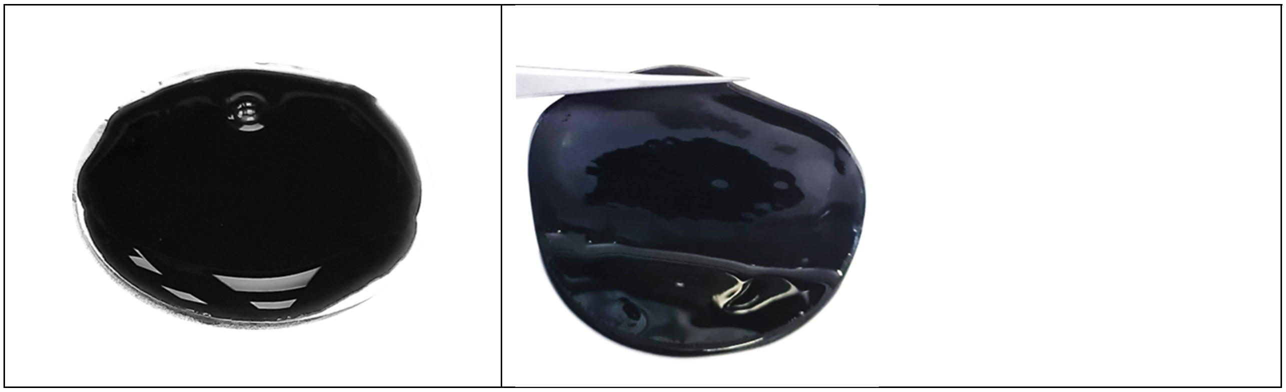

Figure S10– Images of the hydrogel before and after peeling

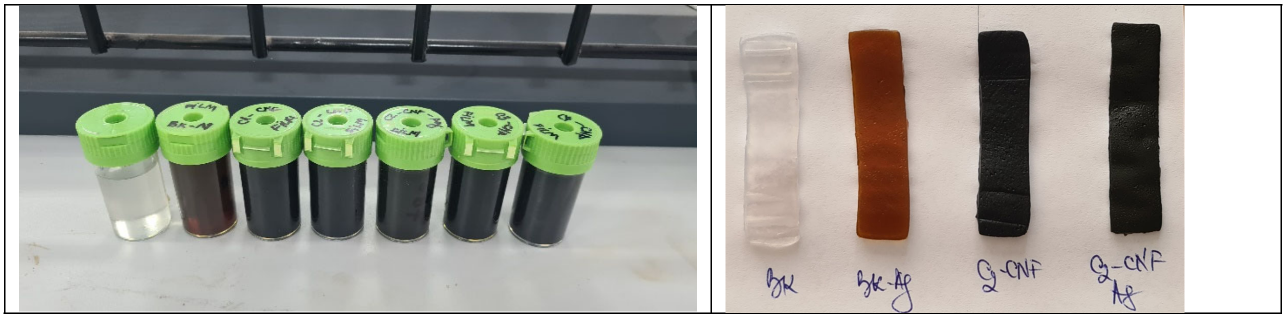

Figure S11 – Images of the decontamination solutions and samples for tensile testing

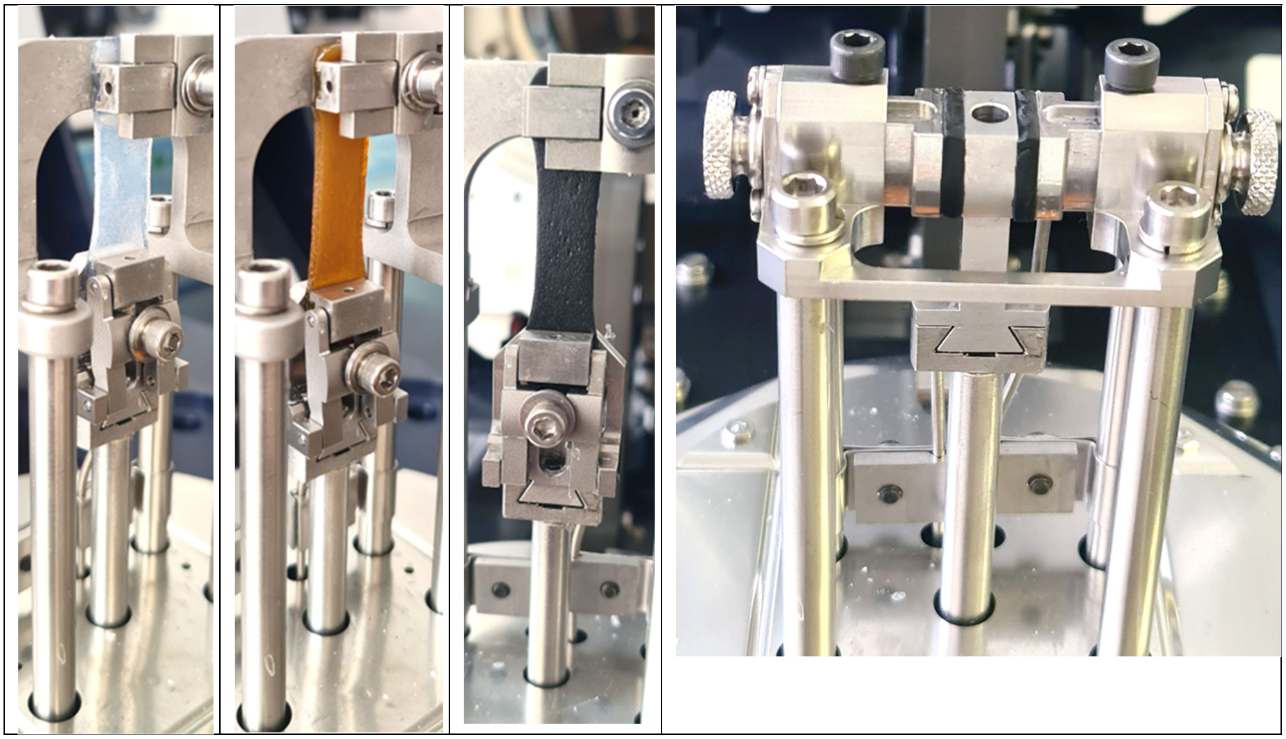

Figure S12 – Images of the hydrogels during mechanical properties characterization

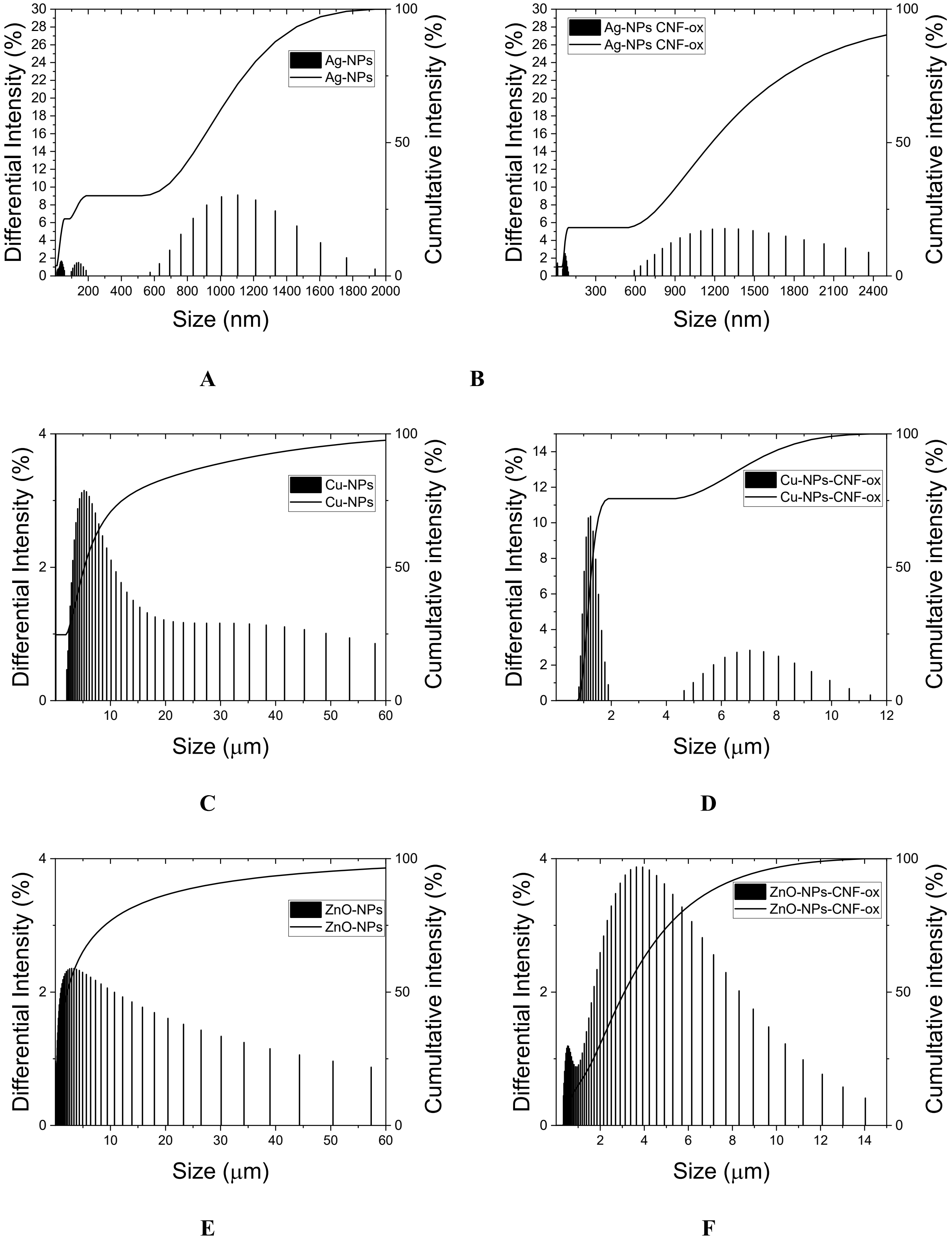

Figure S13 DLS analysis for the nanoparticles and CNF-ox decorated with nanoparticles.

Supplementary materials

Table S2 - Zeta (ζ) potential measurements results

Sample	Zeta (ζ) Potential (mV)
**Ag-NPs**	-36.72
**Ag-NPs-CNF-ox**	-42.5
**Cu-NPs**	-26.80
**Cu-NPs-CNF-ox**	-38.5
**ZnO-NPs**	+28.7
**ZnO-NPs-CNF-ox**	+33.2
